# PLA2R antibodies and PLA2R glomerular deposits in psoriasis patients with membranous nephropathy

**DOI:** 10.1186/s12882-016-0407-3

**Published:** 2016-11-22

**Authors:** Yong-Chun Ge, Bo Jin, Cai-Hong Zeng, Ming-Chao Zhang, Da-Cheng Chen, Ru Yin, Wei-Bo Le

**Affiliations:** National Clinical Research Center of Kidney Diseases, Jinling Hospital, Nanjing University School of Medicine, Nanjing, Jiangsu 210016 People’s Republic of China

**Keywords:** Membranous nephropathy, Psoriasis, PLA2R, Renal biopsy, THSD7A

## Abstract

**Background:**

The association between psoriasis and membranous nephropathy (MN) remains largely unclear. We examined the prevalence of serum PLA2R antibody and characterized the expression of PLA2R and THSD7A in glomeruli in patients with MN and psoriasis.

**Methods:**

A total of 24 patients with MN without evidence of a secondary cause except psoriasis were enrolled. The clinical and pathological features were retrospectively analyzed. Serum anti-PLA2R antibody was measured using IFA Mosaic. Renal tissue samples stored in the laboratory bio-bank were used for PLA2R staining under immunofluorescence microscopy and THSD7A immunohistochemical analysis.

**Results:**

Twenty-four patients (21 male and 3 female) with a mean age of 43.6 ± 15.7 years old were enrolled. Serum anti-PLA2R antibody was positive in 7 patients, which was significantly lower than the positivity observed in idiopathic MN (29.2% vs. 81.7%, *P* < 0.001). Glomerular PLA2R staining was positive in 7 patients with positive serum anti-PLA2R antibody. THSD7A staining was negative in all 24 patients. During the follow-up visits, 13 patients with negative serum PLA2R antibody achieved CR. In contrast, CR was only achieved in 1 patient with positive serum PLA2R antibody, PR was achieved in 2 patients.

**Conclusions:**

The prevalence of serum anti-PLA2R antibody and glomerular expression of PLA2R was significantly lower in patients with psoriasis and MN than in those with idiopathic MN, and THSD7A staining was negative, suggesting that MN is associated with psoriasis in the majority of patients. However, idiopathic MN might also accompany psoriasis in a minority of psoriatic patients with positive serum anti-PLA2R antibody.

## Background

Membranous nephropathy (MN) is a renal disease characterized by subepithelial immune deposits in the glomerulus and is the common cause of nephrotic syndrome in adults. MN has been classified as idiopathic MN and secondary MN associated with other diseases [1]. In 2009, M-type phospholipase A2 receptor (PLA2R) was first reported as a major target antigen for idiopathic MN, and serum autoantibodies to PLA2R can be detected in 70% of patients with idiopathic MN [2]. Thrombospondin type-1 domain-containing 7A (THSD7A) was recently reported as another new target antigen for idiopathic MN, and anti-THSD7A antibodies were positive in the serum of 8-14% patients with idiopathic MN without anti-PLA2R antibodies [3]. Both anti-PLA2R and anti-THSD7A antibodies have been suggested as potential markers for differentiating idiopathic and secondary MN.

Psoriasis is a common chronic inflammatory disorder of the skin, affecting 2% of the population in western countries and 0.47% of the population in China [4–6]. Psoriasis is typically limited to the skin; however, increasing evidence suggests that this condition is associated with systemic disorders, including arthritis, cardiovascular disease, metabolic syndrome, cancer, Crohn’s disease, and diabetes mellitus [7, 8].

An association between kidney disease and psoriasis has also been proposed [9]. A population-based cohort study reported that psoriasis was associated with an increased risk of chronic kidney disease (CKD) independently of traditional risk factors [10]. However, only isolated cases of psoriatic-associated MN have been reported thus far [10–14], and it is not clear whether MN is associated with psoriasis. To our knowledge, there are currently no published studies on the prevalence of serum PLA2R antibodies and the glomerular expression of PLA2R and THSD7A in patients with psoriasis and MN.

In the present study, we evaluated 24 cases of renal biopsy-confirmed MN in patients with psoriasis to examine the prevalence of serum PLA2R antibodies and characterize the glomerular expression of PLA2R and THSD7A.

## Methods

### Study patients

In this retrospective study, we reviewed the records of patients who underwent native renal biopsy between 2003 and 2013 at the National Clinical Research Center of Kidney Diseases, Jinling Hospital, Nanjing University School of Medicine. A total of 33 patients showed biopsy-confirmed MN and psoriasis. Among these individuals, 5 patients with positive anti-nuclear autoantibodies (ANA) and 4 patients with hepatitis B virus (HBV) infection were excluded. A total of 24 patients with MN without evidence of a secondary cause, except psoriasis, were enrolled in the present study. This study was approved through the Ethics Committee of Jinling Hospital, Nanjing University School of Medicine.

### Diagnosis of psoriasis

We reviewed the records of the psoriatic patients to confirm that typical skin lesions of psoriasis had been described, including red macules and papules with adherent silvery scales, the thin film phenomenon, and the dot hemorrhage phenomenon. At least one dermatologist at Jinling Hospital made diagnosed the psoriasis. Psoriasis Area Severity Index (PASI) scores were not available.

### Clinical characteristics

Gender, age, duration of psoriasis and kidney disease, body mass index (BMI), hypertension, and diabetes mellitus were recorded. A BMI ≥25 kg/m^2^ but <28 kg/m^2^ was defined as overweight, and a BMI ≥28 kg/m^2^ was defined as obesity.

Urine protein excretion for 24 hours, urinary sediment red blood cell counts, urinary N-acetyl-β-D glucosaminidase (NAG) enzyme, and urinary retinol-binding protein (RBP) were recorded.

The following blood parameters were also recorded, including serum creatinine; albumin; cholesterol; triglycerides; hemoglobin (Hb; anemia was defined as Hb <12 g/dl in men and <11 g/dl in women); serum C3, C4, and rheumatoid factor (RF); serum ANA, anti-double-stranded DNA antibody (A-dsDNA); serum HBV markers; anti-HCV antibodies; anti-PLA2R antibody, measured using IFA Mosaic (EUROIMMUN AG, Lübeck, Germany); and the CKD Epidemiology Collaboration (CKD-EPI) creatinine equation was used to estimate the glomerular filtration rate (eGFR) [15]. The serum anti-PLA2R antibody was tested using the serum collected at the time of renal biopsy.

### Pathological characteristics

A percutaneous renal biopsy was performed. All cases were processed using light, immunofluorescence and electron microscopy. The renal biopsy procedure was specified as follows: the samples were sectioned at 1.5 μm after embedded in paraffin, followed by hematoxylin-eosin, periodic acid-Schiff, periodic acid-silver methenamine (PASM),and Masson staining. More than 10 glomeruli were observed under light scope in each renal tissue sample. The samples were sectioned under frozen conditions and cut at 4 μm for immunofluorescence staining, followed by staining for IgG, IgA, IgM, C3, C1q, κ and λ-light chain using polyclonal FITC-conjugated antibodies. The deposits, staining intensity, and distribution were observed. The electron microscopy observations were performed using a Hitachi 7500 electron microscope.

The indirect immunofluorescence assay of deposits of IgG subclasses was performed as previously reported [16]. The slides prepared under frozen conditions were dried, sealed with 10% fetal serum, followed by rinsing with PBS for 5 minutes. The primary mouse anti-human IgG1, IgG2, IgG3 and IgG4 monoclonal antibodies (clone 8c/6-39, HP-6014, HP-6050 and HP-6025, Sigma-Aldrich) were diluted 1:400 and added, followed by culturing for 2 hours. FITC-labeled rabbit anti-mouse IgG secondary antibody (1:50; DAKO) was added after rinsing the slides with PBS for 5 minutes, and then cultured for 30 minutes. The slides were dried and sealed with glycerin, and observed under a fluorescence microscope. The immunofluorescence intensity of the IgG subclasses was graded as negative, 1+, or 2 + .

PLA2R staining was performed for all patients using the renal tissue samples stored in our bio-bank. The biopsies were stained for subsequent immunofluorescence analysis of the pronase-digested paraffin sections using rabbit anti-PLA2R as the primary antibody (Sigma-Aldrich) and polyclonal goat anti-rabbit IgG (Life Technologies) as the secondary antibody. Glomerular THSD7A expression was observed through immunohistochemical analysis according to the methods of Tomas et al. [3]

### Outcomes

Remission of MN was defined according to 2012 KDIGO guidelines [17]. A complete remission (CR) was defined as a urine protein <0.5g/24h, a partial remission (PR) was defined as a urine protein of 0.5-3.5g/24h with ≥50% reduction compared with baseline. Patients who did not meet the definitions of CR or PR were assigned to no response (NR).

### Statistical analysis

Continuous variables are expressed as the means ± the standard error or median. Differences between the groups were analyzed using Student’s t-test. The qualitative data were analyzed using the chi-square (χ2) or Fisher’s exact test, as indicated and expressed as percentages. The reported p-values were two-sided, and a *p*-value < 0.05 was considered statistically significant. All the analyses were performed using SPSS software (version 18.0, SPSS Inc., USA).

## Results

### General clinical information

The present study enrolled 24 patients (21 male and 3 female) with a mean age of 43.6 ± 15.7 years old (ranging from 17 to 69), a median duration of psoriasis of 72 (4-480) months, and a median duration of kidney disease of 2 (0.3-108) months. All 24 patients presented with psoriasis vulgaris. Psoriasis vulgaris occurred before the onset of kidney disease in 23 patients. Psoriasis vulgaris and MN were diagnosed at the same time in only 1 patient. Interestingly, among the 18 patients with a detailed BMI record, 6 patients were obese and 4 patients were overweight. In addition, 5 of the 24 patients were diagnosed with diabetes mellitus, 9 patients were diagnosed with hypertension, and 3 patients were diagnosed with anemia. The general characteristics of the patients at renal biopsy are listed in Table 1.

**Table 1:** Clinical findings at biopsy and follow-up visits in patients with psoriasis and membranous nephropathy

Case	Gender	Duration of psoriasis(m)	Duration of kidney disease(m)	Upro (g/24 h)	RBC (×10^4^/ml)	Serum Alb (g/L)	SCr (mg/dl)	eGFR (ml/min/1.73 m^2^)	Anti-PLA2R antibody	Treatment	Outcome
1	M	36	36	3.95	1	29.7	0.64	143.95	-	Pred + TwHF	CR
2	M	60	4	7.71	1	29.9	0.87	107.95	-	TwHF	CR
3	M	12	2	2.9	1	37.9	0.85	122.82	-	TwHF	CR
4	F	96	48	1.11	1	39.3	0.73	118.94	-	FK506	CR
5	M	24	2.33	5.97	35	21	0.61	138.79	-	TwHF,CsA	CR
6	M	36	12	0.74	1	45.9	0.9	109.49	-	TwHF	CR
7	F	84	2	5.44	1	21.2	0.52	126.83	-	NA	LOST
8	M	60	2	4.78	1	21.9	1.31	55.16	-	NA	LOST
9	F	120	3	0.59	1	38.7	0.48	128.39	-	Pred + TwHF	CR
10	M	12	2	9.3	1	25.9	0.73	114.41	-	TwHF	CR
11	M	180	6	7.43	1	24.6	1.31	64.38	-	Pred + TwHF	CR
12	M	4	1.33	4.91	34	23.5	0.71	100.55	+	TwHF	ESRD
13	M	12	1	4.24	1	24.1	1.09	89.33	+	Pred + TwHF	PR
14	M	120	0.33	4.96	1	21.7	0.81	112.74	-	Pred + TwHF	CR
15	M	240	108	8.29	1	25.4	1.01	81.04	+	TwHF	PR
16	M	24	2	2.5	1	37.9	0.68	110.58	-	TwHF	CR
17	M	216	2	0.63	1	44.9	0.85	108.23	-	TwHF	CR
18	M	360	1	1.09	27	37.7	0.91	89.38	+	TwHF	CR
19	M	48	3	5.23	1	30.7	0.8	122.43	+	Pred + TwHF	NR
20	M	120	15	9.42	1	17.8	2.18	31.97	+	Pred + TwHF	NR
21	M	60	0.3	8.42	74	19.2	1.55	51.43	+	NA	LOST
22	M	240	0.25	5.47	8	20.5	1.12	76.18	-	FK506 + Pred	CR
23	M	480	1	8.67	10	19.8	1.02	75.7	-	NA	LOST
24	M	480	4	6.02	1	18.8	2.09	32.94	-	NA	LOST

All patients were negative for ANA, A-dsDNA and RF. C3 was decreased in 4 patients, and C4 was normal in all patients. The urinary markers of renal tubular injury were also observed. NAG enzyme was increased in 20 patients, and RBP was increased in 14 patients.

### Pathologic manifestation

**Fig. 1:**
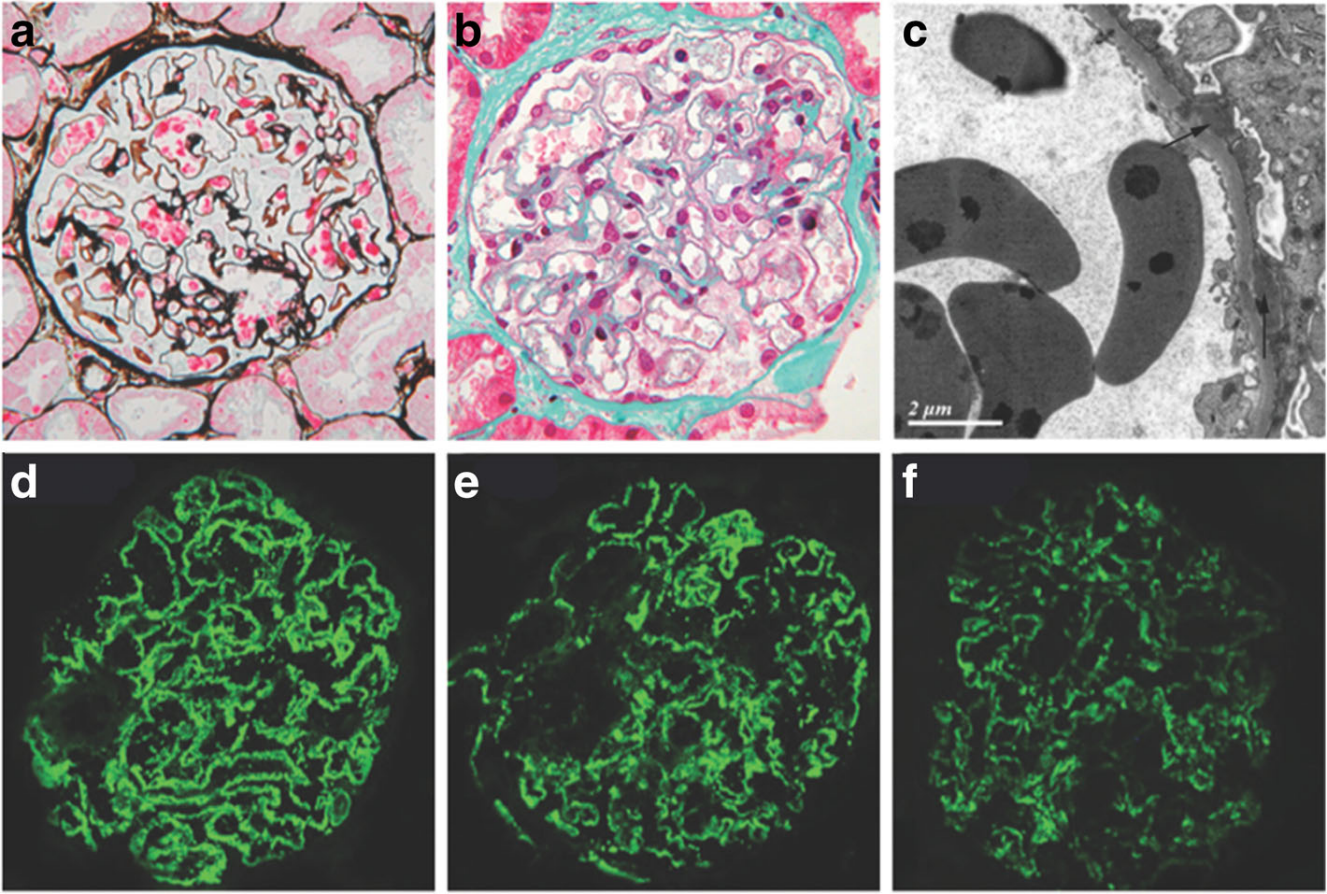
Pathologic findings of MN in patients with psoriasis. (**a**) and (**b**) Glomeruli with subepithelial fuchsinophilic deposits along the epithelium (PASM staining and Masson trichrome; original magnification × 400). (**c**) Glomerular subepithelial electron-dense deposits (*arrow*) with foot process effacement (electron micrograph). (**d**)-(**f**) Staining for IgG (2+), C3 (2+) and C1q (1+) along the glomerular basement membrane (immunofluorescence staining; original magnification, ×400)

The pathological characteristics of the patients are shown in Fig. 1. Stiff glomerular peripheral capillary loops, subepithelial fuchsinophilic deposits, and thickening of glomerular basement membrane were observed under light microscopy. Atypical MN with mesangial proliferation was observed in 23 patients (mild proliferation in 20 patients and moderate in 3 patients). A total of 16 patients showed a few infiltrating cells in glomeruli, including monocytes and/or neutrophil granulocytes, and 15 patients had mild tubulointerstitial fibrosis, 13 patients had acute tubular injury, and 18 patients had focal concentrations of infiltrated monocytes, neutrophil granulocytes and plasma cells. Hyaline degeneration of the interstitial small artery was observed in 12 patients (9 patients with hypertension and 3 patients without).

The findings from immunofluorescence and electron microscopy analyses are listed in Table 2. Granular deposits of IgG and C3 along the capillary loop of the glomeruli were observed in all the patients. Codeposits of C1q were positive in 11 patients. Positivity for IgG, IgA, IgM, C3, and C1q were present in 4 patients. The immunofluorescence staining of IgG subclasses was also performed. Deposits of IgG1, IgG2, IgG3 and IgG4 were observed in 4 patients, deposits of IgG1, IgG2 and IgG4 were observed in 13 patients, and deposits of IgG1 and IgG4 were observed in 6 patients. In the 7 patients with PLA2R positive MN, 5 patients had IgG4 predominant staining in biopsy, 1 patient had equal fluorescence intensity of IgG1 and IgG4 staining, and 1 patient had no glomeruli under immunofluorescence staining.

**Table 2:** IF and EM findings in patients with psoriasis and membranous nephropathy

Case	IF findings							Electron microscopy findings			
	IgG	IgA	IgM	C3	C1q	PLA2R	IgG subtyping	Subepithelial deposits	Subendothelial deposits	Mesangial deposits	Stage of MN
1	1+	1+	1+	1+	1+	Neg	IgG1 1+ , IgG2 1+, IgG4 2+	ND	ND	ND	ND
2	2+	1+	Neg	2+	Neg	Neg	IgG1 2+ , IgG2 1+, IgG4 1+	ND	ND	ND	ND
3	1+	Neg	1+	1+	1+	Neg	IgG1 2+, IgG4 2+	ND	ND	ND	ND
4	2+	1+	1+	2+	Neg	Neg	IgG1 2+, IgG4 2+	ND	ND	ND	ND
5	2+	1+	Neg	2+	Neg	Neg	IgG1 2+ , IgG2 1+, IgG4 2+	+	Neg	Neg	2
6	4+	1+	1+	2+	1+	Neg	IgG1 2+, IgG4 1+	+	Neg	Neg	2
7	2+	Neg	Neg	1+	Neg	Neg	IgG1 2+ , IgG2 1+, IgG4 2+	+	+	+	2
8	2+	Neg	Neg	2+	1+	Neg	IgG1 2+ , IgG2 1+, IgG4 1+	+	Neg	Neg	2
9	2+	1+	Neg	1+	Neg	Neg	IgG1 2+, IgG4 2+	+	Neg	+	3
10	2+	Neg	Neg	2+	1+	Neg	IgG1 2+ , IgG2 1+, IgG4 2+	+	Neg	+	1
11	2+	Neg	Neg	2+	1+	Neg	IgG1 2+ , IgG2 1+, IgG4 1+	+	+	+	1
12	2+	1+	1+	2+	1+	+	IgG1 1+ , IgG2 1+, IgG4 2+	+	Neg	Neg	3
13	2+	Neg	Neg	1+	1+	+	IgG1 1+, IgG2 1+, IgG3 1+, IgG4 2+	+	Neg	+	3
14	2+	Neg	Neg	2+	Neg	Neg	IgG1 2+, IgG4 1+	+	Neg	Neg	1
15	2+	Neg	Neg	1+	Neg	+	No glomeruli	+	Neg	Neg	3
16	2+	Neg	Neg	Neg	Neg	Neg	IgG1 1+ , IgG2 1+, IgG4 2+	+	Neg	+	2
17	2+	1+	1+	1+	1+	Neg	IgG1 2+ , IgG2 1+, IgG4 1+	+	Neg	+	3
18	2+	Neg	Neg	2+	1+	+	IgG1 1+ , IgG2 1+, IgG4 2+	+	Neg	Neg	2
19	2+	2+	Neg	1+	Neg	+	IgG1 2+ , IgG2 1+, IgG4 2+	+	Neg	+	3
20	2+	Neg	Neg	2+	1+	+	IgG1 2+, IgG2 1+, IgG3 1+, IgG4 3+	+	Neg	Neg	2
21	2+	Neg	Neg	2+	Neg	+	IgG1 1+, IgG2 1+, IgG3 2+, IgG4 2+	+	+	+	3
22	2+	Neg	1+	2+	Neg	Neg	IgG1 2+, IgG4 2+	+	Neg	Neg	2
23	2+	Neg	Neg	2+	Neg	Neg	IgG1 3+, IgG2 2+, IgG3 1+, IgG4 2+	+	Neg	Neg	1
24	2+	Neg	Neg	2+	Neg	Neg	IgG1 2+ , IgG2 1+, IgG4 2+	+	Neg	Neg	3

The detailed characteristics were recorded under electron microscopy in 20 patients, showing that 4 patients were classified as stage I MN, 8 patients were classified as stage II MN and 8 patients were classified as stage III MN. In addition to subepithelial electron-dense deposits, electron-dense deposits in the mesangium were observed in 9 patients. The effacement of foot processes in podocytes was observed in all patients.

### Serum anti-PLA2R antibody and glomerular expression of PLA2R and THSD7A

Serum anti-PLA2R antibody was positive in only 7 of the 24 patients, a result significantly lower than that observed in idiopathic MN in a previous report (29.2% vs. 81.7%, *P* < 0.001) [18].

**Fig. 2:**
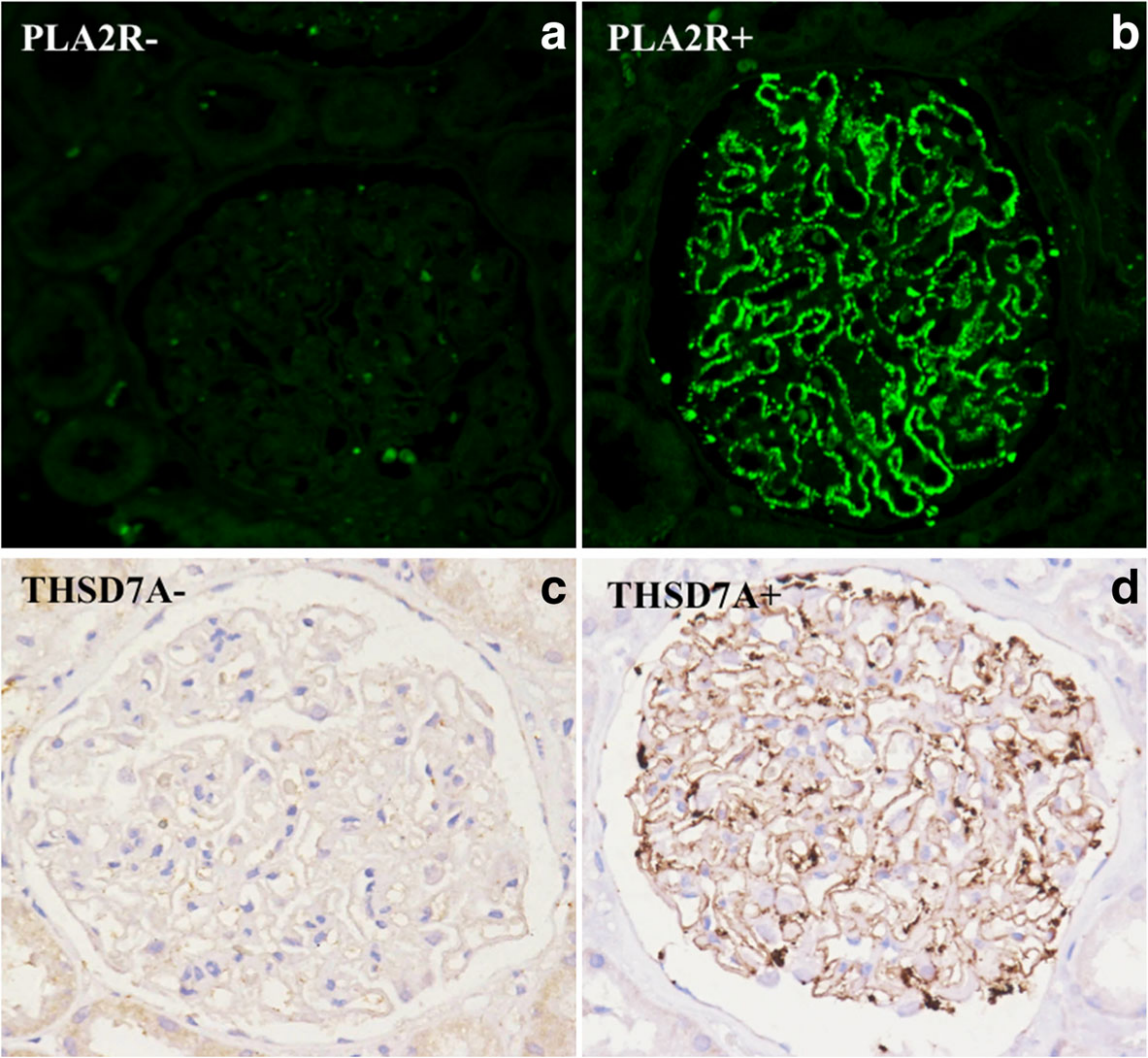
Expression of PLA2R and THSD7A in glomerular observed under immunofluorescence microscopy. Staining for PLA2R was negative in 17 patients (**a**), and positive in 7 patients with MN and psoriasis (**b**). THSD7A was negative in all 24 patients through immunohistochemical analysis (**c**), and a positive control is shown in patients with idiopathic MN (**d**)

The glomerular expression of PLA2R and THSD7A was observed through immunofluorescence staining and immunohistochemical analysis, respectively (Fig. 2). PLA2R was positive in the glomeruli of 7 patients whose serum PLA2R antibody was positive, which is significantly lower than that in patients with idiopathic MN, as reported previously [19] (29.2% vs 69.3%, *P* = 0.001). THSD7A was negative in all 24 patients.

We compared the clinical characteristics between patients with serum PLA2R antibody and patients without PLA2R antibody. There was no significant difference in proteinuria, serum albumin, serum creatinine, and eGFR.

### Treatment and prognosis

The treatments for psoriasis prior to renal biopsy included oral steroids in 3 patients, and oral Tripterygium wilfordii Hook F (TwHF) in 2 patients. The remainder of the patients had used topical treatments, such as corticosteroids, vitamin D analogs, and sulfur ointments; no NSAIDs, cyclosporin A, methotrexate,D-penicilliamine, or gold salts were used in these patients.

Treatment and prognosis information for MN was available for 19 patients, and 5 patients were lost during the follow-up visits (Table 1). A total of 10 patients were treated with TwHF, while 7 patients were treated with TwHF plus prednisone (Pred) at 30 mg per day, and 2 patients were treated with tacrolimus.

CR was achieved in 14 (73.7%) patients (8 patients were treated with TwHF, 4 patients were treated with TwHF plus Pred, and 2 patients were treated with tacrolimus). PR was achieved in 2 (10.5%) patients (1 patient was treated with TwHF and 1 patient was treated with TwHF plus Pred). NR was observed in 3 (15.8%) patients who received TwHF plus Pred, including 1 patient who progressed to end stage renal disease after 12 months treatment with TwHF.

Interestingly, we also observed that in the 17 patients with negative serum PLA2R antibody, 13 patients achieved CR and 4 patients were lost. In contrast, in the 7 patients with positive serum PLA2R antibody, CR was achieved in only 1 patient, PR was achieved in 2 patients, NR was in 3 patients, and 1 patient was lost during the follow-up visits. Unfortunately, the prognosis of psoriasis was not quantitative evaluated because PASI scores were not available at baseline and follow-up visits. However, we indeed observed the significant improvements in skin lesions in patients 2, 10, 11, 16, who achieved CR in proteinuria after the TwHF or Pred + TwHF treatment. The detailed outcomes of psoriasis in other patients were not available during follow-up visits.

## Discussion

MN is classified as either idiopathic or secondary to various underlying diseases, such as autoimmune diseases, viral hepatitis, malignancies, or exposure to toxins or drugs. The M-type PLA2R and THSD7A have been identified as major target podocyte antigens involved in adults with idiopathic MN, and serum anti-PLA2R antibodies have a high sensitivity and specificity for idiopathic MN [2, 3, 18, 20–22]. Although MN associated with psoriasis has also been documented in case reports, describing improvements in both MN and skin lesions in psoriatic patients and the complete remission of proteinuria and skin symptoms after the successful treatment of psoriasis, there have been no studies concerning the glomerular expression of PLA2R and THSD7A in patients with psoriasis and MN. Thus, the association of psoriasis and MN remains uncertain.

The present study was the first to examine the prevalence of serum PLA2R antibodies and characterize the glomerular expression of PLA2R and THSD7A in patients with psoriasis and MN. The findings showed only 7 patients with positive serum anti-PLA2R antibody and glomerular PLA2R expression, which was significantly lower than that observed in patients with idiopathic MN [18]. The anti-PLA2R antibody was measured using IFA Mosaic in the present study, while it was measured using western blot in the previous study. Although different assays may have different sensitivity and specificity, a series of studies have confirmed the concordant results of anti-PLA2R antibody with IFA Mosaic and western blot methods [23–25]. In addition, we also perform the PLA2R staining of the renal tissue. Hence, the results of our present study were reliable and comparable to the results of our previous reported study. These findings also indicated that the MN was secondary in a majority of patients with psoriasis. However, the coincidental occurrence of idiopathic MN with psoriasis should be considered in the patients with serum anti-PLA2R antibody.

Although psoriasis is a common chronic inflammatory disorder of the skin, increasing evidence has demonstrated that psoriasis is associated with an increased risk of CKD and urinary albumin excretion [26, 27]. A population-based cohort study demonstrated that moderate to severe psoriasis was associated with an increased risk of CKD, independent of traditional risk factors [10]. In another cross sectional study, Yang et al reported that renal failure was more prevalent in patients with severe psoriasis than in age- and sex-matched controls [28]. In addition, multiple cross-sectional studies had also observed a greater prevalence of microalbuminuria [26]. Psoriatic nephropathy is recently described. Nephropathy associated with psoriasis has been increasingly reported in recent years. These kidney diseases include IgA nephropathy [29, 30], MN [11–14], membranoproliferative glomerulonephritis [31], focal segmental glomerulosclerosis [32], minimal change disease [33], AA-amyloidosis [34], and therapy-related tubular-interstitial alteration [26].

In the present study, 24 psoriatic patients were diagnosed with MN according to renal pathological findings and had long-standing psoriasis prior to the appearance of proteinuria. These patients were never administered nephrotoxic drugs (such as NSAIDs, gold salts, or D-penicillamine) or experienced systemic lupus erythematosus (SLE),malignancies,or HBV or HCV infection. However, immunofluorescence assays showed that 11 of the 24 patients had granular parietal C1q deposits associated with IgG in the glomeruli, and 4 of these 11 patients had codeposits of IgA and IgM. C1q is typically absent or observed at low levels in idiopathic MN, whereas it is typically more present in secondary disease [35]. Thus, it is reasonable to speculate that the pathogenesis of MN in psoriatic patients differs from the pathogenesis of idiopathic MN.

Additionally, immunofluorescence staining showed that variations in the distribution of IgG subclasses reflect predominant Th1 and Th2 immune responses [36]. Larsen et al had found that secondary membranous nephropathy with positive PLA2R1 showed IgG4-predominant staining, the IgG4 predominance raises the possibility that these cases are more pathogenically related to primary membranous nephropathy than secondary [37]. In this study, glomerular deposits of both IgG1 and IgG4 were observed in all patients, indicating that both Th1 and Th2 immune response participated in the pathogenesis of MN associated with psoriasis. The immunofluorescence intensity of IgG1 was stronger than that of IgG4 in 7 of 17 patients with negative serum PLA2R antibody, in contrast the intensity of IgG4 was stronger than that of IgG1 in 5 of 7 patients with positive serum PLA2R antibody. The distribution of glomerular IgG subclasses further indicated that MN was secondary in cases of psoriasis with negative serum PLA2R antibody, and idiopathic MN might also be coincident with the occurrence of psoriasis in patients with positive serum PLA2R antibody.

The optimal therapeutic management of secondary MN involves treating the underlying clinical conditions and diseases implicated in the etiology of MN. In the present study, the response to treatment with Pred and (or) TwHF was better in patients with negative PLA2R antibody than in those with positive PLA2R antibody. Previous studies have also demonstrated that proteinuria was decreased after the successful treatment of psoriasis. The relatively well response to treatment with corticosteroids and TwHF also indicated that MN was associated with psoriasis in patients with negative PLA2R antibody.

The mechanism underlying the association between MN and psoriasis remains unclear. Some authors have suggested that the immunological mechanism responsible for the association between SLE and secondary MN could also be involved in psoriasis [12, 14]. Susceptibility loci shared between patients with psoriasis and SLE in a Chinese population has been identified [38]. In addition, the circulation antigen or immune complexes associated with psoriasis could also deposit in the subepithelial space as another potential mechanism for MN associated with psoriasis. Unfortunately, no antigen associated with psoriasis and MN has been identified because of the relatively rare cases. In the present study, although 5 patients with positive ANA were excluded, low C3 levels were observed in 4 patients, implying that an autoimmune mechanism might play an important role in the association between MN and psoriasis.

Psoriasis vulgaris was observed in all the 24 patients,which is the most common psoriasis type in China [5]. Increasing evidence suggests that psoriasis is associated with diabetes, metabolic syndrome, and cardiovascular disease, independent of traditional risk factors [39–41]. The results of the present study also indicated that a high prevalence of obesity, diabetes mellitus and hypertension as additional risk factors, consistent with the findings of a previous study.

Due to its retrospective nature, this study has several limitations. First, the severity of psoriasis was not recorded; therefore, potential associations between the severity and renal manifestations of psoriasis could not be analyzed. Second, the treatments and outcomes of skin lesions were not quantitative analyzed during the follow-up visits. Although these limitations exist, the findings of the present study suggest that dermatologists and nephrologists should be aware of the association between MN and psoriasis.

## Conclusions

In summary, we observed the prevalence of serum PLA2R antibodies and glomerular expression of PLA2R and THSD7A in patients with psoriasis and MN. The lower proportion of positive serum PLA2R antibody and glomerular expression of PLA2R, negative expression of THSD7A, pathological manifestation and distribution of IgG subclasses indicated that MN was associated with psoriasis in a majority of patients. However, idiopathic MN might also be coincident with the occurrence of psoriasis in patients with positive serum PLA2R antibody. Thus, it is important for dermatologists and nephrologists to be aware of the association between membranous nephropathy and psoriasis. Further studies are needed to determine the pathogenesis, optimal treatment, and outcomes of membranous nephropathy associated with psoriasis.

## Abbreviations

A-dsDNA: Anti-double-stranded DNA antibody
ANA: Anti-nuclear autoantibodies
BMI: Body mass index
CKD: Chronic kidney disease
CR: Complete remission
eGFR: estimate glomerular filtration rate
HBV: Hepatitis B virus
MN: Membranous nephropathy
NAG: N-acetyl-β-D glucosaminidase
NR: No remission
PASI: Psoriasis Area Severity Index
PASM: Periodic acid-silver methenamine
PLA2R: M-type phospholipase A2 receptor
PR: Partial remission
Pred: Prednisone
RBP: Retinol-binding protein
RF: Rheumatoid factor
THSD7A: Thrombospondin type-1 domain-containing 7A
TwHF: Tripterygium wilfordii Hook F
